# Arabidopsis PARG1 is the key factor promoting cell survival among the enzymes regulating post-translational poly(ADP-ribosyl)ation

**DOI:** 10.1038/srep15892

**Published:** 2015-10-30

**Authors:** Hailei Zhang, Zongying Gu, Qiao Wu, Lifeng Yang, Caifeng Liu, Hong Ma, Yiji Xia, Xiaochun Ge

**Affiliations:** 1 State Key Laboratory of Genetic Engineering, Institute of Plant Biology, Department of Biochemistry and Molecular Biology, School of Life Sciences, Fudan University, Shanghai 200438, China; 2 Department of Biology, Hong Kong Baptist University, Kowloon Tong, Kowloon, Hong Kong, China

## Abstract

Poly(ADP-ribosyl)ation is a reversible post-translational modification of proteins, characterized by the addition of poly(ADP-ribose) (PAR) to proteins by poly(ADP-ribose) polymerase (PARP), and removal of PAR by poly(ADP-ribose) glycohydrolase (PARG). Three PARPs and two PARGs have been found in Arabidopsis, but their respective roles are not fully understood. In this study, the functions of each PARP and PARG in DNA repair were analyzed based on their mutant phenotypes under genotoxic stresses. Double or triple mutant analysis revealed that PARP1 and PARP2, but not PARP3, play a similar but not critical role in DNA repair in Arabidopsis seedlings. PARG1 and PARG2 play an essential and a minor role, respectively under the same conditions. Mutation of PARG1 results in increased DNA damage level and enhanced cell death in plants after bleomycin treatment. *PARG1* expression is induced primarily in root and shoot meristems by bleomycin and induction of *PARG1* is dependent on ATM and ATR kinases. PARG1 also antagonistically modulates the DNA repair process by preventing the over-induction of DNA repair genes. Our study determined the contribution of each PARP and PARG member in DNA repair and indicated that PARG1 plays a critical role in this process.

In mammals such as human and mouse, a type of enzyme called poly(ADP-ribose) polymerase (PARP) can recognize and bind to the single or double strand DNA breaks in the genome and become activated^1^,^2^,^3^. PARPs use nicotinamide adenine dinucleotide (NAD^+^) as a substrate to attach the ADP-ribose moiety onto protein acceptors. The successive attachment of ADP-ribose residues produces long and branched poly(ADP-ribose) chains which are linked to glutamate, aspartate or lysine residues of the target proteins^4^, resulting in the poly(ADP-ribosyl)ation modification of proteins. PARPs are the primary substrates of themselves and the poly(ADP-ribosyl)ated (PARylated) PARPs recruit proteins important for DNA repair to the damaged sites, facilitating the DNA repair process^1^,^5^.

Later studies found that PARPs are also involved in other physiological processes, including chromatin remodelling, transcriptional regulation, ubiquitinylation regulation, spindle and centrosome function and stress granule formation^4^,^6^,^7^, in addition to DNA repair. PARPs are located in both the nucleus and cytoplasm^8^. The PARylated proteins can recruit PAR binding proteins, such as XRCC1, DNA ligase III, KU70, DNA-PK, ALC1, and APLF, and these proteins may also be PARylated by PARPs^9^,^10^.

So far, most of the knowledge about the cellular functions of poly(ADP-ribosyl)ation comes from animal systems. There are 17 PARP members in human and hPARP1 and hPARP2 are the most extensively studied^4^,^11^. They are localized in nucleus and involved in DNA repair. Other PARPs are mostly localized in cytoplasm and carry out functions other than DNA repair^8^. Among the hPARP proteins, only 6 are considered to be bona fide PARPs, including hPARP1 and hPARP2. Others are either mono(ADP-ribosyl) transferases or inactive proteins^4^,^11^.

Arabidopsis has three PARP members. All PARP enzymes have been shown to be located in nucleus^12^,^13^,^14^. Inhibition or silencing of PARPs improves abiotic stress tolerance, enhancing resistance to drought, high light, heat and oxidative stresses^15^,^16^,^17^, and perturbs innate immune responses to microbe-associated molecular patterns such as flg22 and elf18^18^, resulting in a compromised basal defense response^13^,^19^. Chemical inhibition of Arabidopsis PARP activity enhances plant growth and reduces anthocyanin accumulation^20^,^21^. PARP1 and PARP2 are involved in microhomology mediated end joining (MMEJ) during DNA repair process^22^, and a recent report indicated that PARP2 is the predominant PARP in Arabidopsis DNA damage and immune responses^13^. PARP3, unlike PARP1 and PARP2, lacks the conserved HYE triad important for PARP catalytic activity^4^,^11^, and is mainly expressed in developing seeds^12^. It is reported that PARP3 is necessary for maintaining seed viability during storage^12^. Whether it is involved in DNA repair during post-germination stage remains unknown.

PARGs catalyze the reverse reaction of poly(ADP-ribosyl)ation by breaking the ribose-ribose linkage in the ADP-ribose polymers^23^. PARGs are widely found in bacteria, filamentous fungi, animals and plants. In human, mouse and fly, a single *PARG* gene is found, which produces different isoforms by alternative splicing. These isoforms may exist in different subcellular locations and take part in different cellular processes^24^. Loss-of-function of PARG results in embryonic lethality in mouse and causes larval-stage death in *Drosophila melanogaster*^25^,^26^. In *C. elegans*, two *PARG* genes, *PME-3* and *PME-4* have been reported. They are mainly expressed in nerve cells. Silencing of each or both of them induces a hypersensitivity to ionizing radiations but has no obvious developmental effects^27^.

Two tandemly-arrayed *PARG* genes, *PARG1* and *PARG2*, are found in Arabidopsis. They share 57% identity and 68% similarity in amino acid sequence. A recent report indicated that PARG1 is an active enzyme and PARG2 is not^19^. The PARG-signature motif GGL-X6-8-QEE is important for the PARG activity^23^,^28^. PARG1 contains the canonical PARG motif while PARG2 has an amino acid substitution in the motif, which is GGL-X_6-8_-QEE. They both have the tyrosine clasp, which is necessary for substrate binding in mammalian PARGs^29^. In addition, a G450R point mutation of Arabidopsis PARG1 beyond these two motifs has been shown to inactivate PARG1 and leads to enhanced immune gene expression^19^. The *parg1* mutant in Arabidopsis is sensitive to the microbe-associated molecular pattern elf18 and to the DNA cross-linking agent MMC^29^, and also has reduced tolerance to drought, osmotic, and oxidative stresses^30^. Furthermore, PARG1 plays a role in regulating Arabidopsis circadian rhythm and in the photoperiod-dependent transition from vegetative growth to flowering^31^. So far no function has been assigned to PARG2.

Although the roles of PARP1 and PARP2 in DNA damage signaling have been reported, how PARPs and PARGs contribute to and coordinate this process remains elusive. DNA damage signals are mainly transduced by two sensor kinases: ATM (Ataxia telangiectasia mutated), which mediates double strand break (DSB) signaling, and ATR (ATM and Rad3-related), which responds to single strand breaks (SSB) and DNA replication stress^32^. These two kinases coordinately regulate most of the DNA damage responses in animals and plants. ATM phosphorylates SOG1 (suppressor of gamma response 1)^33^, which is the master transcription factor regulating DNA damage response in plants^32^,^34^ and is a functional counterpart of animal p53 although it has no structural similarity to p53^32^. Through activation of SOG1, cells undergo DNA repair, cell-cycle arrest, programmed cell death or endoreduplication^32^. It is interesting to understand how poly(ADP-ribosyl)ation affects the DNA damage response.

Emerging evidence shows that poly(ADP-ribosyl)ation may have a unique mode of action in plants^12^,^13^,^29^,^31^, but the detailed genetic and biochemical analyses are lacking to address the various phenotypes in different biological processes, especially the functional relevance between PARPs and PARGs in DNA repair. In this study, we compared the knock-out mutant phenotypes of all *PARPs* and *PARGs* in Arabidopsis under the same conditions. We found that PARG1 is the key enzyme mediating DNA damage response. It promotes cell survival under genotoxic stress and regulates the DNA damage response by avoiding over-induction of DNA repair genes.

## Results

### Phenotypic comparison of the loss-of-function mutants of all *PARP* and *PARG* genes in Arabidopsis

There are three *PARP* genes and two *PARG* genes in Arabidopsis. To reveal the critical gene(s) for DNA repair in poly(ADP-ribosyl)ation, we obtained all knock-out mutants of these genes from different sources. The T-DNA insertion sites in *parp1*, *parp2* and *parp3* mutants are listed in Supplementary Fig. S1. These mutants have been used in previous studies^12^,^13^,^19^,^22^. For *PARG1* and *PARG2*, two mutants for each gene were used, respectively. *parg1-4* and *parg2-2* are mutants reported for the first time. Therefore, the T-DNA insertions in these mutants were confirmed by genomic PCR, and expression of the mutant allele was examined by reverse transcription (RT)-PCR (Supplementary Fig. S2). The results indicated that they are null mutants of *PARG1* and *PARG2*, respectively.

**Figure 1:**
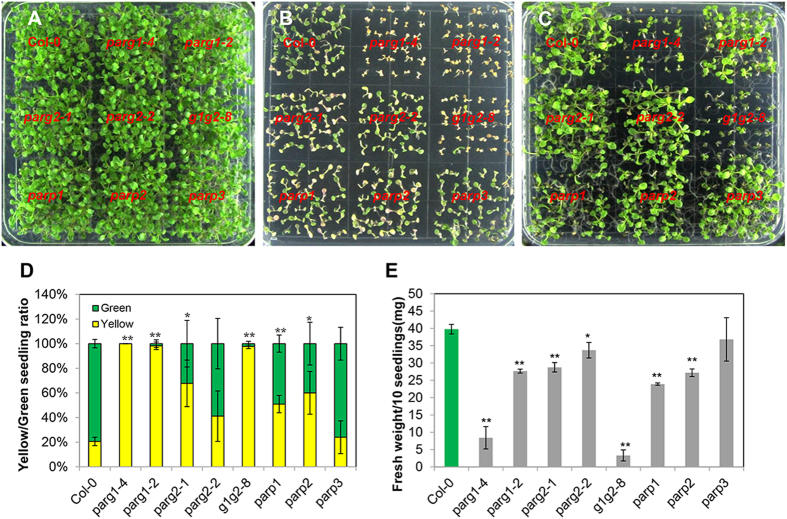
Phenotypes of the knock-out mutants of *PARP* and *PARG* genes in Arabidopsis. (**A**) 1/2 MS plate. (**B**) 1/2 MS plate with 50 μg ml^−1^ bleomycin. (**C**) 1/2 MS plate with 100 μg ml^−1^ MMS. The seedlings were photographed after grown for two weeks. (**D**) Comparison of the yellow/green seedling numbers of each mutant grown on bleomycin plates. (**E**) Fresh weight per 10 seedlings of each mutant grown on MMS plates. All experiments were done for at least three times and similar results were obtained. The data were presented as means of three replicates ± SE. Significant differences (t-test) compared to Col-0 under the same conditions are indicated by asterisks: *P < 0.05; **P < 0.01.

We grew these mutants on the same plate to compare their phenotypes. Two kinds of genotoxic agents, bleomycin and methyl methane sulfonate (MMS), were used in the assays, respectively. Bleomycin is a glycopeptide which mainly induces DSBs^35^ and MMS is a monofunctional alkylating agent that induces N-alkyl lesions and SSBs^36^. DSBs and SSBs can activate different DNA repair pathways^37^,^38^. DSBs are the most severe damage type since chromosomal breakage can lead to cell death^32^,^38^. On control plates, all mutants grew normal and were indistinguishable from each other (Fig. 1A). On the plate with 50 μg/ml bleomycin, *parg1-2* and *parg1-4* turned almost completely yellow (Fig. 1B,D), while *parp1, parp2* and *parg2-1* were only mildly sensitive to bleomycin compared with Col-0, and *parg2-2* and *parp3* responded similarly as the wild type control (Fig. 1B). MMS blocked the growth of parg*1-2* and *parg1-4*, but did not induce yellowing (Fig. 1C), so we measured the fresh weight of each mutant and compared them with that of the Col-0 (Fig. 1E). Most of the single mutants exhibited slight sensitivity to MMS except *parp3*, which had no obvious phenotype, and *parg1-4*, which was hypersensitive to MMS. Surprisingly, another mutant allele of *PARG1*, *parg1-2*, is only slightly sensitive to MMS (Fig. 1C,E).

Taken together, the results revealed that among all mutants, the *parg1-4* mutant displayed the most severe phenotype while *parp3* had no obvious phenotype compared to the wild type control when challenged by DNA SSB or DSB inducing agents.

### Phenotypic analysis revealed the relationships between PARP and PARG family members

**Figure 2:**
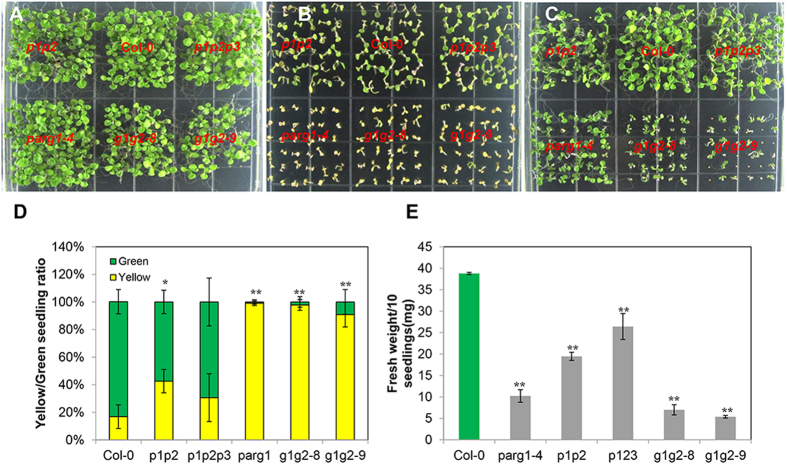
Phenotypic comparison of the double or triple mutants generated for the *PARP* or *PARG* gene family. (**A**) 1/2 MS plate. (**B**) 1/2 MS plate with 50 μg ml^−1^ bleomycin. (**C**) 1/2 MS plate with 100 μg ml^−1^ MMS. (**D**) Comparison of the yellow/green seedling numbers of each mutant grown on bleomycin plates. (**E**) Fresh weight per 10 seedlings of each mutant grown on MMS plates. All experiments were done for at least three times and similar results were obtained. The data were presented as means of three replicates ± SE. Significant differences (t-test) compared to Col-0 under the same conditions are indicated by asterisks: *P < 0.05; **P < 0.01.

It has been reported that among the three *PARPs*, when *PARP1* or *PARP2* is absent, the expression levels of the other two *PARPs* are up-regulated^12^. We also found that when *PARG1* is knocked out, the remaining *PARG2* gene is up-regulated, and vice versa (Supplementary Fig. S3A). Therefore, functional redundancy might exist between the same family members. To further analyze it, we constructed *parp1parp2* (*p1p2*) double and *parp1parp2parp3* (*p1p2p3*) triple mutants. We were unable to generate double mutant of the closely linked *PARG1* and *PARG2* genes, so RNA interference was used to inhibit the expression of *PARG2* in the *parg1-4* mutant background (*g1g2*). All these mutants grew normally on 1/2 MS plate (Fig. 2A), while phenotypic comparison by bleomycin and MMS treatments revealed that the *p1p2p3* triple mutant was not more sensitive than the *p1p2* double mutant (Fig. 2B–E), indicating that either PARP3 does not perform the same role as PARP1 and PARP2, or is not an active enzyme. This speculation is also supported by the analysis result of the *parp3* single mutant, which was not sensitive to bleomycin and MMS. Two *g1g2* lines, *g1g2-8* and *g1g2-9*, were more sensitive to bleomycin and MMS than wild type control (Fig. 2B,C). Inhibition of *PARG2* expression in the *parg1-4* mutant did not enhance the sensitivity of *parg1-4* to bleomycin, which was already severe, but did enhance its resistance to MMS (Fig. 2B,C).

The phenotype of the *g1g2* mutant was much more severe than that of the *p1p2* and *p1p2p3* mutants on both bleomycin and MMS plates (Fig. 2B,C), indicating a more detrimental effect of PARG mutation to DNA repair than that of PARPs mutation. Between the two PARGs, PARG1 is the major one determining plant growth and survival under bleomycin and MMS treatments. We therefore focused on the study of PARG1 and mainly used *parg1-4* mutant for the following experiments due to its consistently strong phenotype on bleomycin and MMS plates. When a genomic fragment spanning from 1379 bp upstream of the translational start site to 969 bp downstream of stop codon of *PARG1* was introduced into *parg1-4*, the bleomycin- and MMS-sensitivity phenotype of *parg1-4* were rescued (Supplementary Fig. S4), demonstrating that loss of function of *PARG1* caused the phenotypes. To simplify the analysis, we used bleomycin to induce DNA damages in the following experiments.

### PARG1 degrades poly(ADP-ribose) *in vitro*

PARG is considered as the downstream enzyme of PARP, but disruption of *PARG1* caused more severe phenotype than that of *p1p2* double and *p1p2p3* triple mutant under genotoxic stress. It raised the question whether the phenotype is directly caused by the loss of PARG activity. To answer this question, we first examined the poly(ADP-ribose) glycohydrolase activity of PARG1 *in vitro* using recombinant PARG1 protein. We cloned the cDNA of PARG1 and expressed PARG1 as a glutathione-S-transferase (GST) fusion protein in *E. coli*. The purified GST-PARG1 was used for the activity assay.

**Figure 3:**
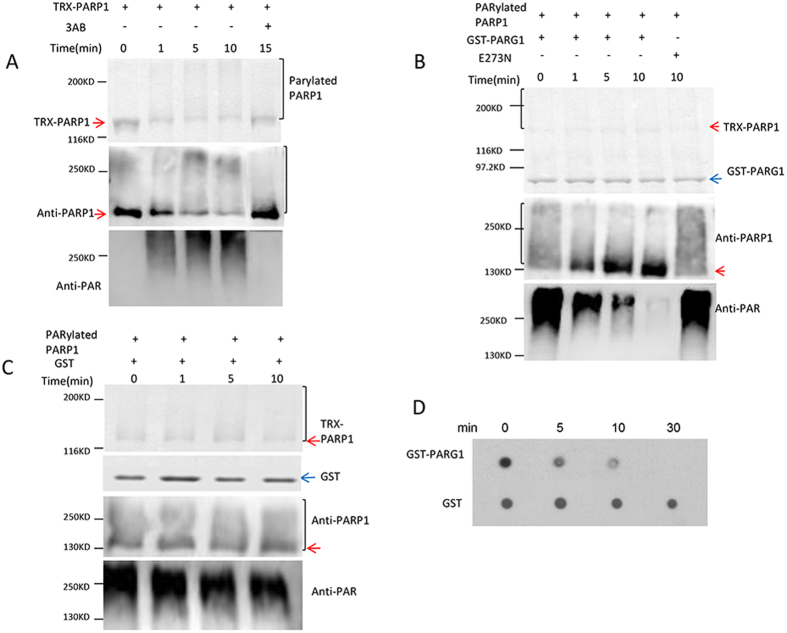
PARG1 has PAR-degrading activity. (**A**) Generation of the PARylated PARP1 substrate. Auto-modified PARP1 protein migrated as a smeared band on SDS-PAGE gel. Anti-PARP1 and anti-PAR antibody were used for detection of the corresponding PARP1 protein and PAR on the proteins. (**B**) PARylated PARP1 substrate was incubated with the recombinant GST-PARG1 or mutated protein E273N for different time periods, and then subjected to SDS-PAGE analysis. Anti-PARP1 and anti-PAR antibody were used for tracking the changes of PARP1 protein and PAR on proteins. (**C**) PARylated PARP1 substrate was incubated with GST tag protein for different time periods, and then subjected to SDS-PAGE analysis. Anti-PARP1 and anti-PAR antibody were used for tracking the changes of PARP1 protein and PAR on proteins. (**D**) GST-PARG1 and GST protein were incubated with commercial PAR, and then subjected to immune-dot blot analysis by anti-PAR antibody.

Arabidopsis PARP1 can be self-modified by adding varying numbers of ADP-riboses onto itself in the presence of broken DNA and NAD^+^^14^. The auto-PARylated protein was detected as an upwardly shifted smear on SDS-PAGE gel (Fig. 3A upper and middle panel). This auto-modified PARP1 was used as a PARylated protein substrate for the PARG1 activity assay. PAR was detected by anti-PAR antibody (Fig. 3A bottom panel). This antibody tends to recognize big ADP-ribose polymers due to their stronger immunogenicity. When the auto-modified PARP1 was incubated with the recombinant GST-PARG1 protein, the upward smear of the PARP1 disappeared and the band became concentrated again at the original size of PARP1 on the SDS-PAGE gel within one minute of adding GST-PARG1 (Fig. 3B middle panel), and PAR signal on the protein was concomitantly reduced (Fig. 3B bottom panel), indicating that PARG1 can rapidly degrade the poly(ADP-ribose) on the modified PARP1 and restore the size of the protein (Fig. 3B middle panel). For comparison, a mutant protein E273N (Glu273 → Gln) with a mutation in the conserved GGG-X6-9-QEE motif of PARG1 was generated and subjected to the same assay. The mutation completely destroyed the PAR hydrolase activity (Fig. 3B). As a control, the GST tag did not cause any mobility change of the PARylated PARP1 (Fig. 3C). When the commercial PAR was incubated with the recombinant PARG1 protein for different time periods and then detected with anti-PAR antibody, the PAR signal also disappeared gradually (Fig. 3D). These results demonstrated that PARG1 has a PAR hydrolase activity not only against PARylated proteins, but also against free PAR.

### Loss of PARG1 induces cell death under genotoxic stress

**Figure 4:**
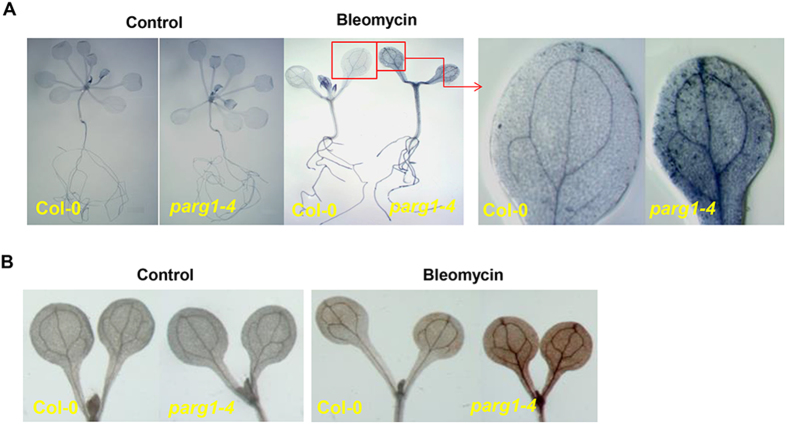
The *parg1-4* seedling leaves show more cell death and accumulate more H_2_O_2_ than that of Col-0 seedlings under genotoxic stress. (**A**) Trypan blue-stained three week-old Col-0 and *parg1-4* seedlings grown on the plates without (control) or with 24 μg ml^−1^ bleomycin. (**B**) DAB stained cotyledons of one week-old Col-0 and *parg1-4* seedlings grown on the plates without (control) or with 24 μg ml^-1^ bleomycin.

When non-lethal dose of bleomycin was included in the medium, the *parg1-4* mutant grew more slowly and had fewer true leaves than wild type seedlings, reflected by a lower fresh weight of *parg1* (Supplementary Fig. S5A,B). Leaf color of the *parg1* mutant turned yellow gradually with the increase of bleomycin concentration (Supplementary Fig. S5C), suggesting that chlorophyll was degraded and cell death might happen. Trypan blue was used to stain the tissues for detection of cell death^39^. The staining results indicated that bleomycin induced more cell death in the leaves of *parg1* than those of wild type (Fig. 4A). Furthermore, stronger H_2_O_2_ accumulation could be detected in the mutant cotyledons (Fig. 4B) when stained by 3, 3’-diaminobenzidine (DAB), which yields a brown precipitate in the presence of H_2_O_2_^40^. Thus, the genotoxin treatment also led to a more oxidized status in the mutant than in wild type.

**Figure 5:**
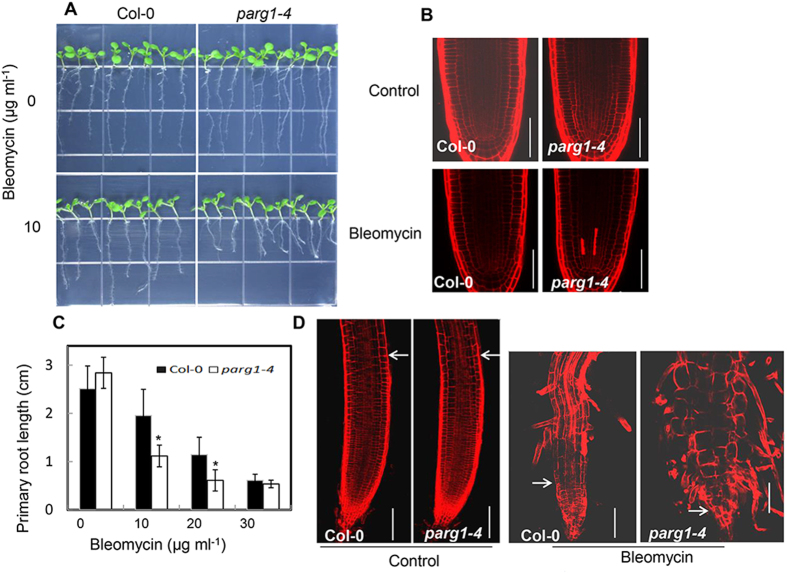
The *parg1-4* mutant root is more sensitive to bleomycin than that of Col-0. (**A**) Comparison of the primary root length of Col-0 and the *parg1-4* mutant under normal condition and 10 μg ml^−1^ bleomycin treatment. (**B**) Propidium iodide-stained root tips of Col-0 and *parg1-4* seedlings grown on plates without (control) or with 10 μg ml^−1^ bleomycin for 4 days. The completely reddish-stained cells indicate the dead cells. Scale bars = 75 μm. (**C**) Statistical analysis of the primary root lengths of Col-0 and the *parg1-4* mutant grown on plates with different concentrations of bleomycin for 10 days. Significant differences (t-test) compared to Col-0 under the same conditions are indicated by asterisks: *P < 0.05; **P < 0.01. (**D**) Propidium iodide-stained root tips of Col-0 and *parg1-4* seedlings grown on plates without (control) or with 20 μg ml^−1^ bleomycin for 10 days. The arrows indicate the boundary of meristematic zone at the root tip before and after bleomycin treatment. Scale bars = 100 μm.

In addition, the root growth of *parg1-4* was also investigated. Under normal conditions, the mutant had no significant difference from Col-0 (Fig. 5A). However, bleomycin treatment caused more cell death in the root meristem of the mutant than that of wild type, shown by propidium iodide (PI) staining (Fig. 5B). The cell death led to significant reduction of the primary root growth of the mutant compared to that of wild type (Fig. 5C). When the time period of the genotoxic stress was extended, a much shorter meristematic zone was observed at the root tip of the *parg1-4* mutant (Fig. 5D), which might be due to the enhanced cell death in the *parg1-4* root meristem. Meanwhile, the epidermal cells of the *parg1-4* mutant were also more deformed and enlarged than that of wild type (Fig. 5D).

These results indicated that disruption of PARG1 causes more cell death in both leaves and roots when the plants were treated by bleomycin.

### PARG1 regulates poly(ADP-ribose) level *in vivo*

**Figure 6:**
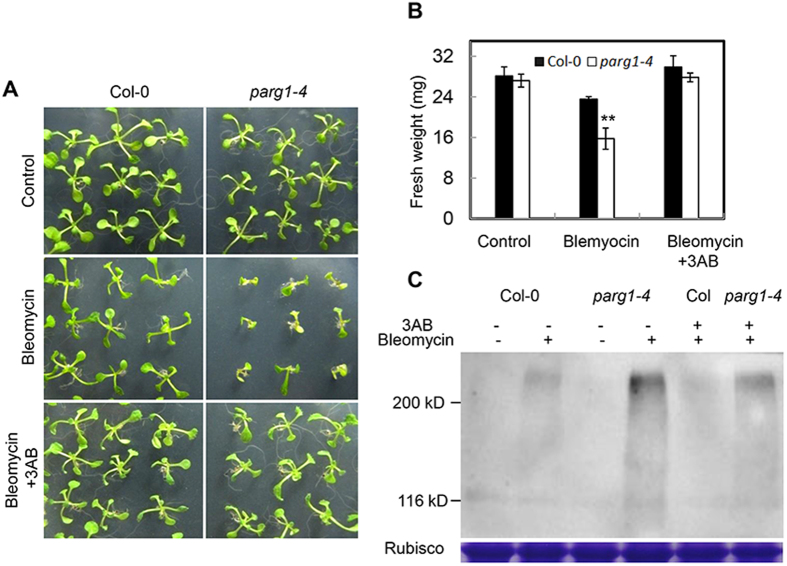
The phenotype of *parg1-4* is caused by the over-accumulation of PAR *in vivo*. (**A**) The phenotype of *parg1-4* can be rescued by the PARP inhibitor 3-AB. The plants were grown on 1/2 MS plate (control), or plates with 25 μg ml^−1^ bleomycin, and 25 μg ml^−1^ bleomycin plus 0.75 mM 3AB, respectively for two weeks. (**B**) Fresh weight per 8 seedlings of the *parg1* mutant grown on the plates described in (**A**). Data were presented as means of three replicates ± SE. Significant differences (t-test) compared to Col-0 under the same conditions are indicated by asterisks: **P < 0.01. (**C**) Detection of poly(ADP-ribosyl)ated proteins in Col-0 and *parg1-4* seedlings grown on plates described in (**A**). RuBisCo large subunit was used to indicate the protein loading amount. This experiment was repeated twice with similar results.

To test whether the phenotype of the *parg1-4* mutant was caused by over-accumulation of poly(ADP-ribose), we added 3-AB, a cell permeable PARP inhibitor^29^ into the media to repress PARP activity. 3-AB reversed the phenotype of *parg1-4* and restored the seedling growth of the *parg1-4* mutant close to that of wild type under bleomycin treatment (Fig. 6A). In addition, the death phenotype of *parg1-4* seedlings under high level of bleomycin was also rescued by 3-AB (Supplementary Fig. S6). These results suggested that PARG1 hydrolyzes PAR synthesized by PARPs *in vivo* and excessive PAR in the *parg1-4* mutant is the reason for growth arrest and cell death under bleomycin treatment. However, *parg1-4* was a little smaller than Col-0 on plates containing bleomycin and 3-AB (Fig. 6A, Supplementary Fig. S6). This may be due to incomplete suppression of PARP activity by 3-AB *in vivo*, or that PARG1 has other functions not affected by 3-AB.

To further confirm the biochemical activity of PARG1 *in vivo*, we examined the PAR levels in *parg1-4* and wild-type plants by western blot analysis using anti-PAR antibody. The results revealed that the levels of PAR were relatively low in both wild type and *parg1-4* plants grown under normal conditions (Fig. 6C). When treated with bleomycin, the *parg1-4* mutant accumulated more PAR than wild type, and 3-AB reduced PAR in both wild type and the *parg1-4* mutant (Fig. 6C). These results once again indicated that PARG1 regulates PAR level *in vivo*.

### PARG1 is induced primarily in mitotically active cells

Quantitative RT-PCR was performed to investigate the expression level changes of *PARG1* under genotoxic stress. The transcript level of *PARG1* in the seedlings subjected to a brief bleomycin treatment was examined (Supplementary Fig. S7). The results showed that *PARG1* expression was induced by bleomycin. GUS staining of *pPARG1::GUS* transgenic line showed that *PARG1* expression was initially induced in the root and shoot meristems (Supplementary Fig. S7B), then extended to other tissues.

### Loss-of-PARG1 leads to the transcriptional up-regulation of DNA repair genes and increase of cellular DNA damage level

**Figure 7:**
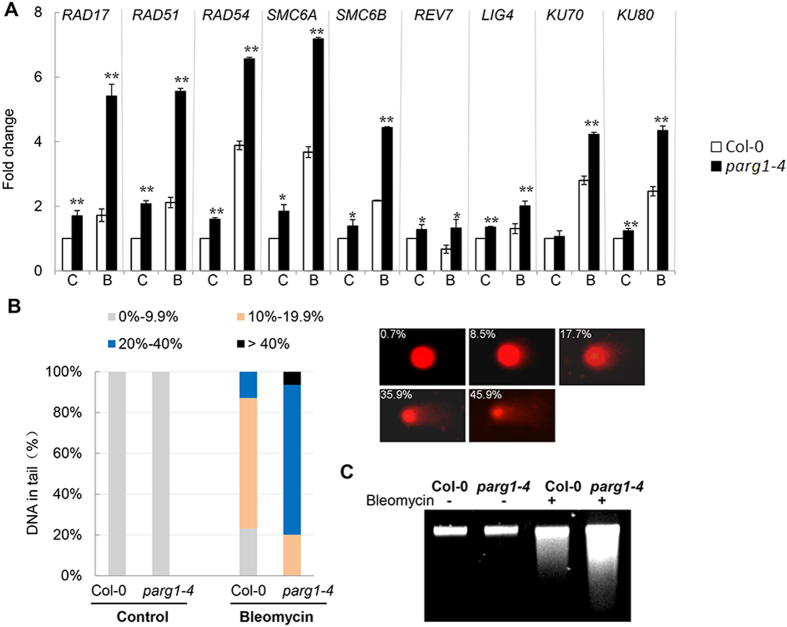
DNA damage level is higher in the *parg1-4* mutant than that in Col-0 plants under genotoxic stress. (**A**) Expression level changes of genes involved in DNA double-strand break repair in roots of Col-0 and *parg1-4* seedlings. The data represent the mean values of three replicates ± SD. (**C**) control; (**B**) bleomycin at 20 μg ml^−1^. (**B**) Comet assay of the DNA damage levels of Col-0 and *parg1-4* seedlings grown on plates with or without (control) 20 μg ml^−1^ bleomycin for 10 days. The percentage of DNA in comet tails was analyzed and quantified by CASP software (http://sourceforge.net/projects/casp/) and used as an indicator of DNA damage level. 100 nuclei for each treatment were randomly selected and imaged. The bar size represents proportion of nuclei falling into the ranges of damage level indicated by different colors, and the images with percentages beside it indicate the examples of damaged nuclei. (**C**) DNA fragmentation assay showed that genomic DNA is more damaged in *parg1-4* than in Col-0.

Eukaryotic organisms have two major pathways for repairing DNA double strand breaks, the homologous recombination (HR) and the non-homologous end joining (NHEJ) pathways^41^,^42^. We examined the expression levels of known genes involved in these two pathways and found that the HR pathway genes *SMC6A*, *SMC6B*^43^, *RAD17*, *RAD51*, *RAD54*^44^ and *REV7*^45^, and the NHEJ pathway genes *LIG4*, *KU70* and *KU80*^46^,^47^, were all up-regulated in the *parg1-4* mutant compared to that in wild type after bleomycin treatment (Fig. 7A). These results suggested that the *parg1* mutant cells might experience more DNA damage than wild type. To test this, we used the comet assay to observe DNA damages in wild type and mutant plants, where the proportion of the comet tail to the head represents the degree of DNA double-strand breaks in the nucleus^48^. No striking changes could be observed in the *parg1-4* mutant under normal growth conditions. However, after treatment with bleomycin, the mutant showed a significantly higher level of DNA damage than wild type plants (Fig. 7B). When the genomic DNA was isolated and subjected to agarose electrophoresis, the DNA of *parg1-4* migrated as a band with more smear than that of Col-0, indicating the presence of severe DNA damages in *parg1-4* (Fig. 7C). Taken together, loss of function of PARG1 compromised the DNA repair process, although the DNA repair genes were still highly induced in the *parg1* mutant.

### Induction of *PARG1* gene is ATM- and ATR-dependent and PARG1 represses the transcriptional up-regulation of *ATM*, *ATR* and *SOG1*

**Figure 8:**
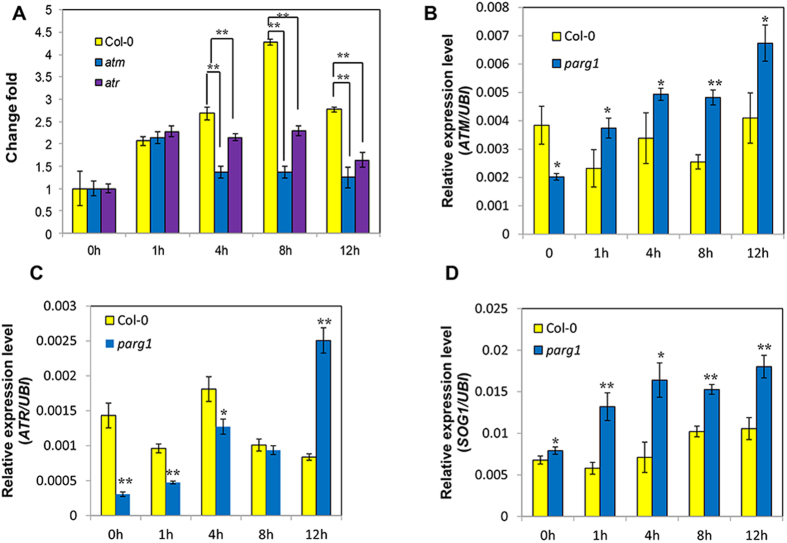
*PARG1* expression is regulated by *ATM* and *ATR* but it antagonistically represses the expression of DNA damage signaling genes. (**A**) Comparison of the induction level of *PARG1* in Col-0, *atm* and *atr* mutants after bleomycin treatment. The fold lines connect the columns for comparison. (**B**–**D**) Comparison of *ATM*, *ATR* and *SOG1* expression levels in Col-0 and in the *parg1* mutant. Seedlings grown for two weeks were treated by 50 μg ml^−1^ bleomycin for different time periods. The data were presented as means of three replicates ± SE. Significant differences (t-test) are indicated by asterisks: *P < 0.05; **P < 0.01.

ATM and ATR are two critical kinases which transduce double and single strand break signals to DNA repair machinery, respectively^32^. They phosphorylate the transcription factor SOG1, which then induce the expression of DNA repair genes^33^,^34^. To understand if PARG1 participates in these two signaling pathways, we compared the expression level of *PARG1* in Col-0, *atm* and *atr* mutants and found that, after bleomycin treatment, *PARG1* expression levels in *atm* and *atr* mutants were obviously lower than that in wild type plants (Fig. 8A), indicating that ATM and ATR are necessary for the induction of *PARG1*. Surprisingly, when the transcript levels of *ATM* and *ATR* in Col-0 and *parg1-4* were compared, *ATM* and *ATR* were both significantly induced in the *parg1* mutant by bleomycin while not in wild type plants within the time periods we examined. The levels of *ATM* in the *parg1* mutant at all time points were higher than those in wild type, except in the untreated plants (Fig. 8B). *ATR* was also induced constantly in the *parg1* mutant, but the beginning level was lower than that of the wild type plants (Fig. 8C). When the expression level of the master transcription factor *SOG1* was examined, it showed a similar up-regulation tendency in the *parg1-4* mutant compared to Col-0 (Fig. 8D). Taken together, mutation of PARG1 caused the transcriptional up-regulation of the key factor genes *ATM*, *ATR* and *SOG1* in plants, suggesting that the expression of PARG1 suppresses the induction of these genes under genotoxic stress.

## Discussion

There is a large family of *PARP* genes but only one *PARG* gene in human and mouse^25^,^26^. The single PARG gene fulfils different functions by generating multiple isoforms^24^. *PARG* gene knock out in mouse causes embryo lethality^25^, indicating an essential role of *PARG* for embryo development. In contrast, there are two *PARG* genes in Arabidopsis, and the *parg1* and *parg2* mutants as well as *parg1parg2*_*RNAi*_ transgenic plants all develop normally under standard growth conditions, suggesting that these genes are not critical for Arabidopsis normal development. However, under genotoxic stress, *PARG1* becomes essential for plant survival. The two knock-out mutants, *parg1-2* and *parg1-4*, are both hypersensitive to bleomycin. Loss of function of PARG1 leads to severe cell death in the mutant after bleomycin treatment, indicating a pivotal role of PARG1 in DNA DSB repair. However, only *parg1-4* is highly sensitive to SSB inducing agent MMS and *parg1-2* is not. This is probably due to that T-DNA in different sites interrupts the gene’s function to different extents. In *parg1-4*, the T-DNA is integrated between Thr311 and Gly312 residues, which are just before the Tyr313 residue in the tyrosine clasp, and the tyrosine clasp is considered important for substrate binding^29^. Therefore, T-DNA in the *parg1-4* mutant may destroy the substrate binding site of PARG1, thus inactivates the protein. In contrast, in the *parg1-2* mutant, T-DNA is integrated between Ala408 and Ser409 residues, which is far from the PARG signature motif (G262 to E274) and also the tyrosine clasp. The *parg1-2* mutant may preserve residual activity of PARG1, which is enough for SSB response, but not for DSB response. As for the *PARG2* gene, both mutants are slightly sensitive to MMS or bleomycin. Inhibition of *PARG2* enhanced the phenotype of the *parg1-4* mutant to MMS, suggesting that *PARG2* does play a role, although minor, in DNA repair. A recent report indicated that PARG2 is an inactive PARG enzyme^19^. However, the expression data that *PARG2* is induced by genotoxin (Supplementary Fig. S3B) and knock-out of *PARG1* leads to a compensatory up-regulation of *PARG2* expression (Supplementary Fig. S3A), together with the phenotypes of *parg2* single mutant and *g1g2* interference plants, all strongly support a role of *PARG2* in DNA repair.

In mouse, the *parp1-/-/parp2-/-* double mutant, like the mouse *parg* mutant, is also embryo lethal^49^. However, the *p1p2p3* triple mutant and *g1g2*_*RNAi*_ mutant in Arabidopsis both develop normally, suggesting that poly(ADP-ribosyl)ation, different from its role in animals, does not play an essential role in Arabidopsis development under standard conditions. Under genotoxic stresses, the phenotypes of the *p1p2* and *p1p2p3* mutants are also weak. Although PARPs can act as detectors for DNA double or single strand breaks, there are other known DNA damage sensors, such as DSB sensor MRN (MRE11/RAD50/NBS1) complex and SSB sensors RPA protein and 9-1-1(RAD9/RAD1/HUS1) complex in plants^32^,^44^. Therefore, loss of functions of three PARPs does not cause disastrous consequence to plants. Furthermore, it is also considered that plants can bear more severe DNA damage than animals due to the immobile nature of cells and no risk for carcinogenesis^32^. By comparing the phenotypes of single, double and triple mutants of Arabidopsis PARP family, we conclude that PARP1 and PARP2 function in the DNA repair process at seedling stage, and PARP3 does not.

Transcriptional studies of the DNA repair genes in the *parg1-4* mutant indicated that the repair pathway is activated, but not succeeded in repairing damage, resulting in the elevation of DNA damage level in the mutant. Over-accumulated DNA damage activates the cell death in the *parg1* mutant, which firstly occurs in the meristems because the meristematic cells are undergoing active DNA replication, giving rise to the high sensitivity of these cells to DNA damaging agents^50^. *PARG1* is primarily induced in root and shoot meristems, consistent with the important role of DNA repair in these cells. Besides cell death, DSBs can also induce endoreduplication in the root tips^51^, which is marked by an enlarged epidermal cell volume. Endoreduplication can prevent the damaged cells from propagation by undergoing DNA replication without cytokinesis. We observed a more pronounced epidermal cell size enlargement effect in the *parg1-4* mutant (Fig. 5D), suggesting that the mutant also undergoes an enhanced reprogramming from mitosis to endocycle besides cell death.

Our expression data showed that *PARG1* acts downstream of both ATM and ATR signaling pathways. ATM and ATR are the major kinases responsible for the activation of DNA repair pathways^32^,^38^,^44^,^52^. They regulate DNA repair process at the post-translational level by phosphorylation of the target proteins such as Chk1, Chk2 in animals and SOG1 in plants^32^,^33^. The induction of *PARG1* is attenuated in both mutants, and the level is even lower in *atm* mutant, indicating a more pronounced effect by the absence of ATM. Interestingly, we found that the lack of PARG1 results in the expression level up-regulation of the key factors *ATM*, *ATR* and *SOG1*, and also other DNA damage responsive genes in plants after bleomycin treatment (Figs 7 and 8). In another word, expression of *PARG1* somehow represses the induction of DNA damage responsive genes. It is recently identified in Arabidopsis that a point mutation of PARG1, which inactivates the PAR hydrolase activity, causes the overexpression of *PR* genes^19^. Therefore, PARG1 probably also regulates the amplitude of stress responses in addition to DNA damage response in plants. In animals, PARPs and PARGs can participate in stress granule formation and AGO (argonaute) proteins can be PARylated by PARPs, therefore relieves microRNA-mediated silencing of target genes^53^. In Arabidopsis, it remains unknown if disruption of PARG1 causes the PARylation level increase of AGOs under stress, thus alleviates the repression of stress-responsive gene by microRNA and leads to transcriptional up-regulation of these genes. Besides this, it is also possible that PAR acts as a stress signal in plants and induces a wide range of stress responses, including DNA damage response. PARG removes PAR so as to reduce the stress signaling. Poly(ADP-ribosyl)ation has been shown to be involved in biotic and abiotic responses in Arabidopsis^13^,^15^,^16^,^19^,^29^,^30^, but the molecular mechanisms are still not very clear until now.

PAR is a death signal in animal cells^54^, which promotes apoptosis-inducing factor (AIF) to translocate from mitochondrion to nucleus to initiate programmed cell death independent of caspase^55^. It is unknown whether this type of cell death exists in plants. The AIF homologue in Arabidopsis, MDAR1, is a monodehydroascorbate reductase mainly localized in peroxisome and involved in removing toxic H_2_O_2_. The *MDAR* genes are involved in multiple stress responses but the function in regulating cell death has not been demonstrated^56^,^57^. Our data indicated that an unknown cell death mechanism may be activated in the *parg1* plants by PAR, since the *parg1* mutant showed cell death phenotype after treatment with bleomycin, and a higher level of PAR is also detected in the mutant. Plants may recognize PAR as a stress signal when it is under a certain threshold but activate cell death when it exceeds the tolerance limit. A better understanding of how cell death is activated in the mutant would contribute to elucidating the molecular mechanism of cell death in plants.

## Methods

### Plant materials and growth conditions

All the Arabidopsis plants used in this study are of the Columbia ecotype. *parp1* (GK_692A05-025067) and *parp2* (SALK_140400C) were provided by de Pater Lab^22^. *parp3* (SALK_108092C), *parg1-2* (SALK_116088) and *parg1-4* (SALK_012110) were ordered from ABRC (Arabidopsis Biological Resource Center, http://www.arabidopsis.org). *parg2-1* (GABI_072B04) and *parg2-2* (GABI_017C05) were from Nottingham Arabidopsis Seed Center (NASC, http://arabidopsis.info). The primers for identification of the new mutants *parg1-4* and *parg2-2* can be found in Supplementary Table S1. Plants were grown at 22 °C under long daylight conditions (12 h/8 h day/night) unless otherwise specified. For seedling phenotype observation, surface-sterilized seeds were germinated on 1/2 MS agar plates with or without bleomycin (Kayaku, Japan) and MMS (Solarbio, China), respectively.

### RNA isolation and quantitative RT-PCR (qRT–PCR)

Total RNA was isolated using TRIzol Reagent (TaKaRa, Japan) and then quantified by measuring OD_260_. Complementary DNA (cDNA) was synthesized using PrimeScript RT-PCR Kit with gDNA eraser (Takara, Japan), and then used for qRT-PCR. PCR were performed with SyBR Premix Ex Taq^TM^ II (TaKaRa, Japan) in a Real One Plus Real-Time PCR System (Applied Biosystem, USA), following the manufacturer’s instructions. Specific primers used can be found in Supplementary Table S1.

### DNA constructs

For *parg1-4* mutant complementation, a genomic DNA fragment of *PARG1* was amplified using PCR (Primers are listed in Supplementary Table S2) and cloned into the *Kpn* I/*Pst* I sites of vector pZP221^58^.

For protein expression in *E.coli*, *PARP1* coding sequence was cloned into pET32a vector (Novagen, USA) in frame with TRX tag using *Sac* I/*Not* I sites. *PARG1* coding sequence was cloned into pGEX-4T-1 vector (GE Healthcare, UK) in frame with GST tag using *EcoR* I/*Sal* I sites (Primers are listed in Supplementary Table S2).

The RNAi construct of *PARG2* was generated by amplifying the nucleotide sequence from 701 to 1140 bp downstream of the start codon of *PARG2* gene, then cloning the fragment into pGEM-RNAi vector in inverted orientations to produce a hairpin fragment spanned by an intron, and finally the hairpin fragment was cloned into p35S-Fast vector^58^.

The construct of *pPARG1::GUS* was generated by amplifying 1140 bp fragment upstream of the start codon of *PARG1* gene and then cloning the fragment into pAKK687 vector using *Xba* I and *BamH* I sites.

### Protein expression and purification

TRX-PARP1 was produced in *E.Coli* Origami (DE3) (Novagen, USA) and GST-PARG1 was produced in Rosetta (DE3) (Novagen, USA). After OD_600_ of the culture reached 0.6, 0.3 mM isopropyl-β-D-thiogalactopyranoside (IPTG) was added into the culture and incubated at 16 °C for 20 h. Bacteria were harvested by centrifuge and then broken by ultra-sonication. The soluble proteins were used for purification. GST and GST-PARG1 were purified using the Glutathione-Agarose affinity resin (Sigma Aldrich, USA) according to the manufacturer’s instructions. TRX and TRX-PARP1 were purified using HisPur Ni-NTA resin (Thermo Scientific, USA).

### Enzymatic activity assays

Preparation of auto-PARylated PARP1 substrate: Purified TRX-PARP1 and TRX proteins were dialyzed completely against 10 mM Tris·HCl pH 7.5 buffer to remove salts. 15 μg TRX-PARP1 or 3 μg TRX proteins were aliquoted into each tube containing 20 mM Tris·HCl pH 7.5, 50 mM NaCl, 7.5 mM MgCl_2_, 0.2 mM DTT, and 10 μM fragmented DNA. To initiate the reaction, NAD^+^ (Sigma Aldrich, USA) was added into each tube to a final concentration of 0.2 mM. The reactions were continued at room temperature for desired time periods and then terminated by adding 300 μl pre-chilled acetone. After centrifugation, the precipitated proteins were subjected to SDS-PAGE and visualized by Coomassie blue staining.

PARG1 activity assay on PARylated proteins: To remove contaminants from poly(ADP-ribosyl)ation reaction, the PARylated TRX-PARP1 substrate was dialyzed against pH7.0 phosphate buffer containing 1 mM 2-mercaptoethanol. Approximately 1 μg PARylated TRX-PARP1 was incubated with 0.2 μg GST or 0.5 μg GST-PARG1 for different time periods in 100 μl dialysis buffer, respectively. The reactions were terminated by the addition of 300 μl ice cold acetone. After centrifugation, the proteins in each reaction were subjected to SDS-PAGE analysis and visualized by Coomassie blue staining. Immuno-blot analysis was performed following standard protocol using anti-PAR (Abcam, UK) or anti-PARP1 antibody (Shanghai Immune Biotech, China) as the primary antibody, respectively. Enhanced chemiluminescence reagents (Thermo Scientific, USA) were used for signal detection.

PARG1 activity assay on free PAR: 0.5 μg GST-PARG1 or GST were incubated with 5 μl of 10 μM PAR (Trevigen, USA) substrate in reaction buffer (25 mM Tris pH 7.5, 150 mM NaCl) at a total volume of 20 μl for different time periods. 2 μl mixture was spotted on nitrocellulose membrane (GE Healthcare, UK). The membrane was allowed to dry at room temperature, then blocked with 1 X TBST plus 5% nonfat milk and detected with anti-PAR antibody.

### Histochemistry observation

Leaf dead cell detection and H_2_O_2_ staining were carried out as described previously^59^. For root dead cell detection, Arabidopsis seedling roots were stained with 10 μg ml^−1^ propidium iodide (Sigma Aldrich, USA). Excitation and emission wavelength were set as 535 nm and 617 nm, respectively for fluorescence microscopy.

### Chlorophyll determination and comet assay

The chlorophyll concentration of seedlings was measured as described^60^. The comet assay was performed based on the method reported before^48^.

### DNA fragmentation assay

One week old seedlings were treated with 10 μg ml^−1^ bleomycin for 48 hrs. Genomic DNA was extracted using the ChargeSwitch gDNA Plant Kit (Invitrogen, USA) and resolved in agarose gel. Each lane was loaded with 100 ng DNA.

### Immuno-blot analysis of PAR in plants

Arabidopsis seedlings were collected and ground in liquid nitrogen, then boiled in 1XSDS-PAGE loading buffer for 5 min and centrifuged. The supernatant was subjected to SDS-PAGE, transferred onto nitrocellulose membrane, and then detected with anti-PAR antibody. Equal loading was monitored by staining the membrane with Coomassie blue solution.

## Additional Information

**How to cite this article**: Zhang, H. *et al.* Arabidopsis PARG1 is the key factor promoting cell survival among the enzymes regulating post-translational poly(ADP-ribosyl)ation. *Sci. Rep.* **5**, 15892; doi: 10.1038/srep15892 (2015).

## Supplementary Material

Supplementary Information
