# Supplementary material for: Arabidopsis PARG1 is the key factor promoting cell survival among the enzymes regulating post-translational poly(ADP-ribosyl)ation
Source: Sci Rep. 2015 Oct 30;5:15892. doi: 10.1038/srep15892 (PMC4626836; doi:10.1038/srep15892)
Supplement: Supplementary Information [file srep15892-s1.pdf]

Arabidopsis PARG1 is the key factor promoting cell survival among the enzymes regulating post-translational poly(ADP-ribosyl)ation

Hailei Zhang[1,3], Zongying Gu[1,3], Qiao Wu[1,3], Lifeng Yang[1], Caifeng Liu[1], Hong Ma[1], Yiji Xia[2] & Xiaochun Ge[1*]

Supplementary Figures and Tables

The following materials are available in the online version of this article:

Figure S1. T-DNA insertion sites in the *parp* mutants used in this study.

Figure S2. Identification of *parg1-4* and *parg2-2* mutants.

Figure S3. Expression levels of *PARG1* and *PARG2* in different plants.

Figure S4. Phenotype of the *parg1-4* mutant under genotoxic stress is caused by the disruption of *PARG1* gene.

Figure S5. Phenotypes of the *parg1-4* seedlings treated by different concentrations of bleomycin.

Figure S6. 3-AB is able to inhibit the death phenotype of the *parg1-4* mutant under severe genotoxic stress.

Figure S7. The *PARG1* expression is induced by genotoxin.

Table S1. Primer list for genomic and RT-PCR.

Table S2. Primer list for DNA constructs.

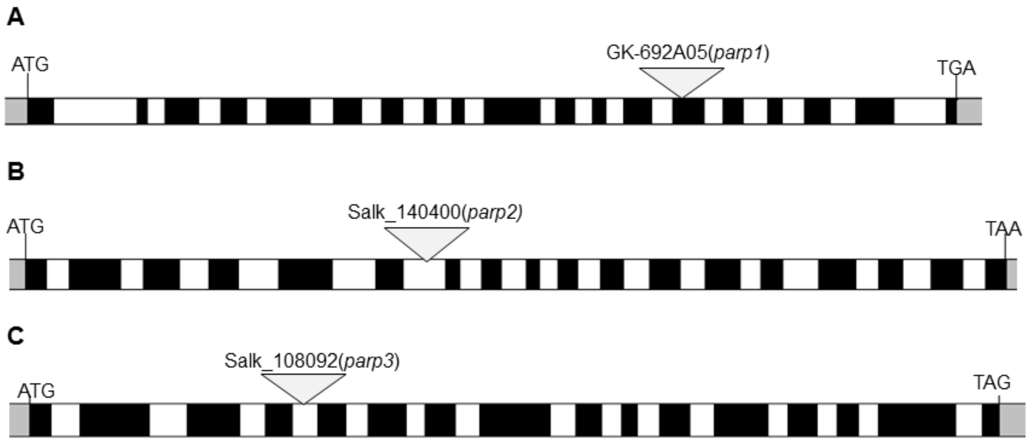

Figure S1. T-DNA insertion sites in the *parp* mutants used in this study. (A) Gene structure of *PARP1* and the T-DNA insertion site of *parp1* mutant. (B) Gene structure of *PARP2* and the T-DNA insertion site of *parp2* mutant. (C) Gene structure of *PARP3* and the T-DNA insertion site of *parp3* mutant. Dark boxes indicate exons and blank boxes indicate introns. Triangles indicate the insertion sites of T-DNA. The light grey triangles indicate the mutants used in this study.

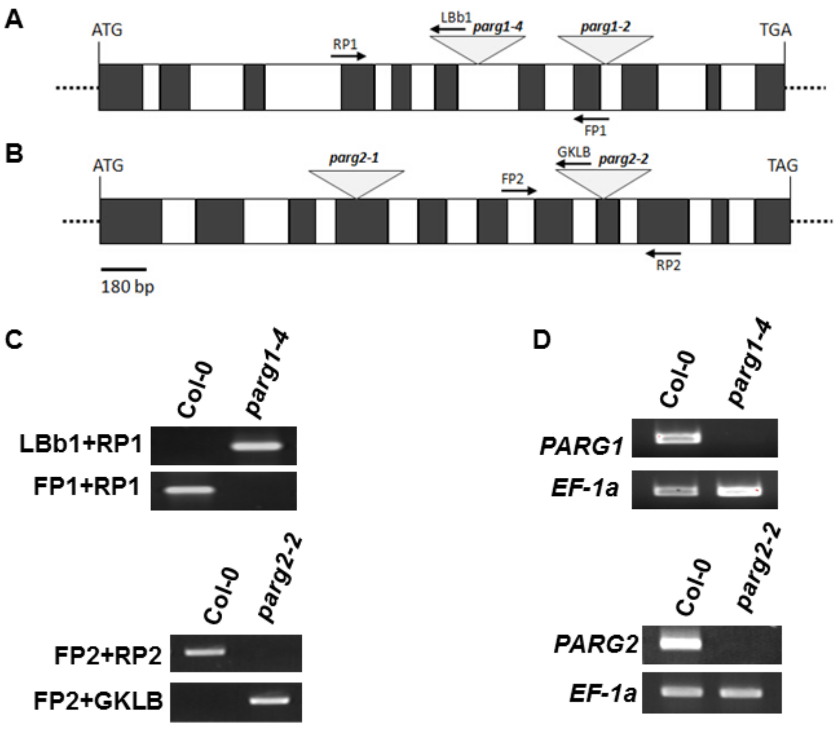

Figure S2. Identification of *parg1-4* and *parg2-2* mutants. (A), (B) Schematic diagram showing the T-DNA insertion sites in the mutants of *PARG1* and *PARG2* gene, respectively. The light grey triangles indicate the mutants used for this study. Arrows indicate the positions of the primers used for identifying mutants. Dark boxes indicate exons and blank boxes indicate introns. (C) Genomic DNA PCR showing that the *parg1-4* and *parg2-2* mutants are homozygous T-DNA insertion lines. (D) RT-PCR showing that *parg1-4* and *parg2-2* are knock-out lines.

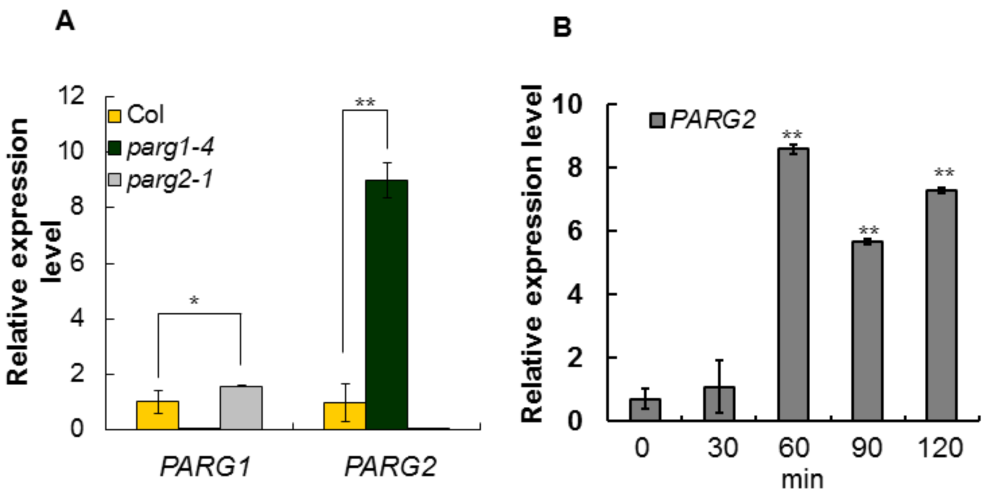

Figure S3. Expression levels of *PARG1* and *PARG2* in different plants. (A) Comparison of the expression levels of *PARG1* and *PARG2* gene in Col-0, *parg1-4* and *parg2-1* mutants. The fold lines connect the columns for comparison. (B) *PARG2* expression is induced by bleomycin in seedlings. Significant differences (t-test) compared to Col-0 are indicated by asterisks: * P < 0.05; ** P < 0.01.

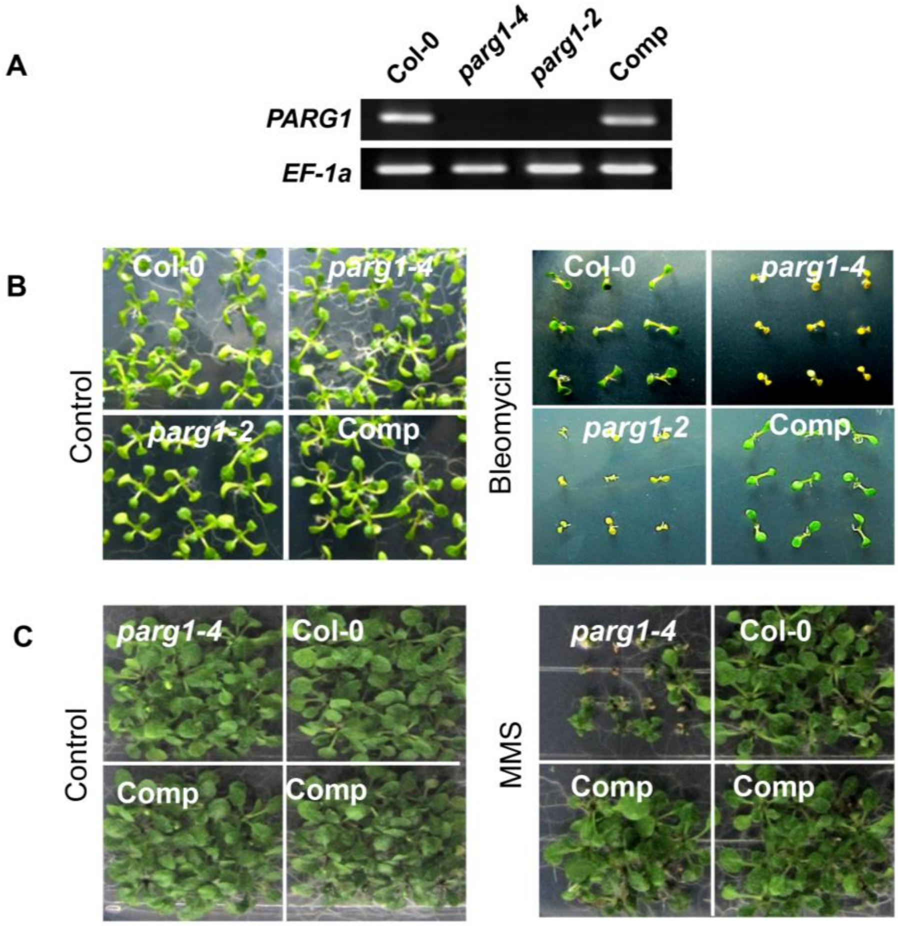

Figure S4. Phenotype of the *parg1-4* mutant under genotoxic stress is caused by the disruption of *PARG1* gene. (A) Expression level of the full length *PARG1* in Col-0, *parg1-4*, *parg1-2* and the complemented line (Comp). (B) Phenotypes of Col-0, *parg1-2*, *parg1-4* and the complemented line grown on 1/2 MS plate (control) and 1/2 MS plate with 50 μg ml^{-1} bleomycin plate, respectively. (C) Phenotypes of Col-0, *parg1-2*, *parg1-4* and the complemented lines grown on 1/2 MS plate (control) and 1/2 MS plate with 100 μg ml^{-1} MMS plate, respectively.

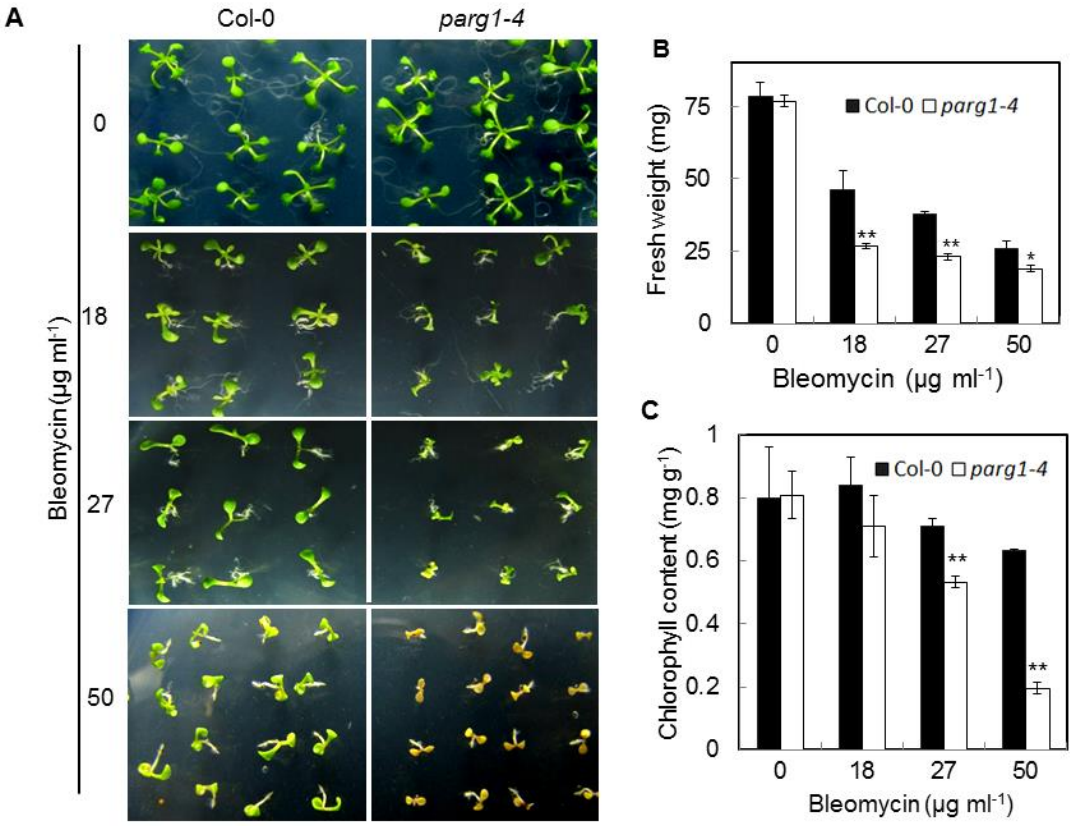

Figure S5. Phenotypes of the *parg1-4* seedlings treated by different concentrations of bleomycin. (A) Phenotypic comparison of Col-0 and *parg1-4* seedlings grown for approximately two weeks on plates containing different concentrations of bleomycin. (B) and (C) Comparisons of fresh weight (B) and chlorophyll content (C) between Col-0 and *parg1-4* seedlings. The fresh weight was determined by weighing 20 seedlings pooled together from each plate. The experiments were done in triplicate and the data were presented as means of three replicates ± SE. Significant differences (t-test) compared to Col-0 under the same conditions are indicated by asterisks: * $P < 0.05$; ** $P < 0.01$.

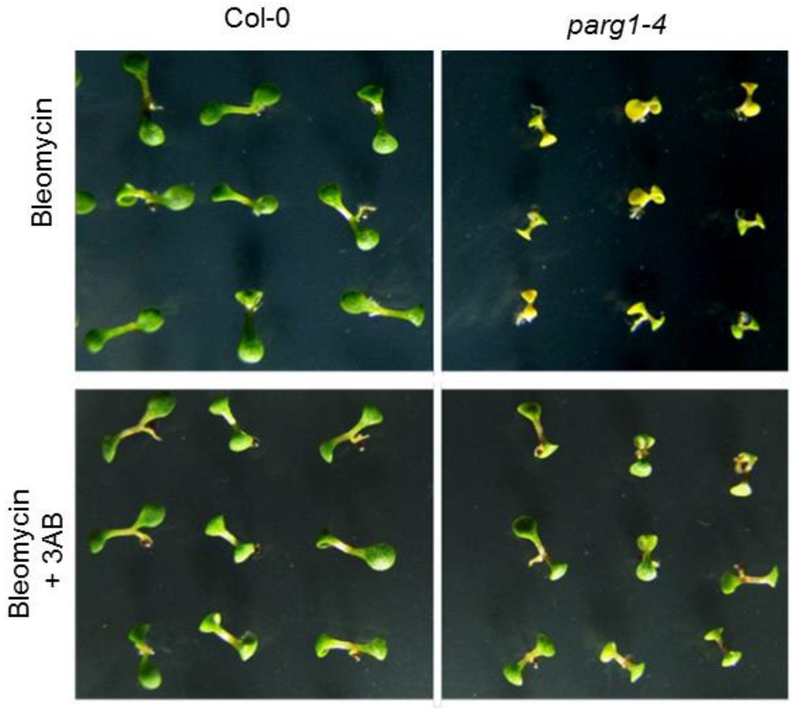

Figure S6. 3-AB is able to inhibit the death phenotype of the *parg1-4* mutant under severe genotoxic stress. Bleomycin is added into plate at 50 μg ml^{-1} and 3AB at 1 mM.

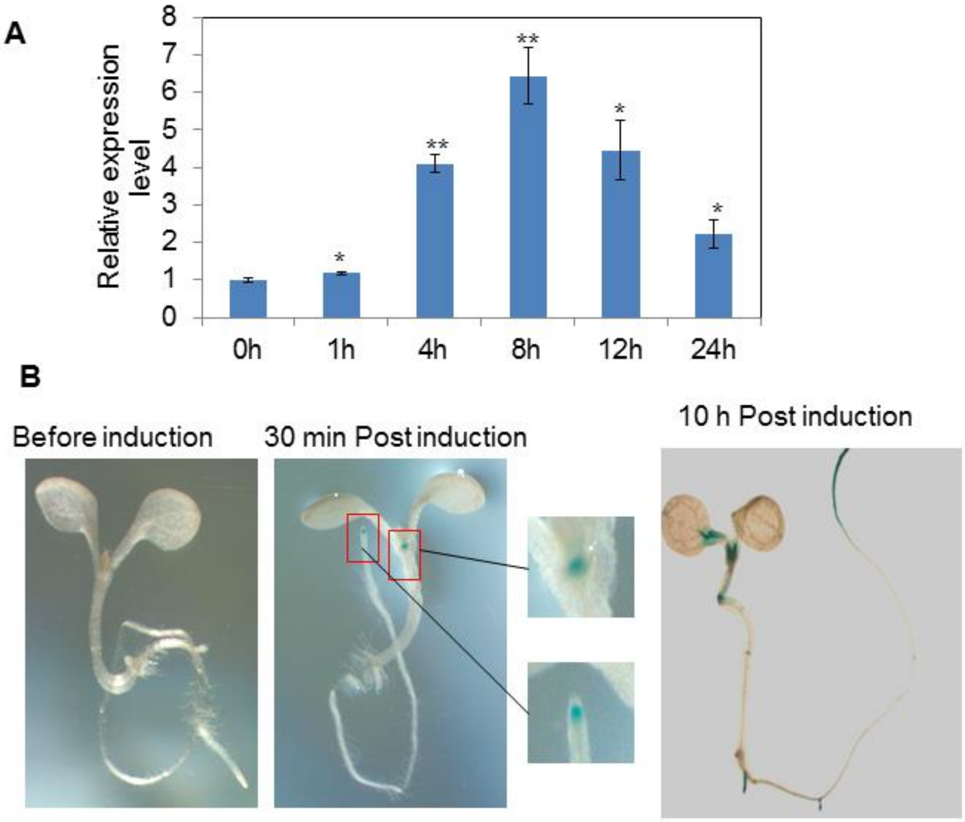

Figure S7. The *PARG1* expression is induced by genotoxin. (A) Examination of the *PARG1* expression level in wild type plants treated by bleomycin for different time. (B) GUS staining of *pPARG1::GUS* transgenic lines indicated that *PARG1* expression is primarily induced in the shoot and root meristems, then extended to other tissues. Significant differences (t-test) compared to Col-0 under the same conditions are indicated by asterisks: * P < 0.05; ** P < 0.01.

Supplementary Tables

Table S1. Primer list for genomic PCR and RT-PCR.

Gene	Forward Primer (5'-3')	Reverse Primer (5'-3')	Usage
Ku70	GGTGTAGCTGCTCCTCGCGC	GCATAGTGTCTCTGCAAAGCGGG	qRT-PCR
Ku80	GCGTCTTGGAGCAGGAGCCAAAG	TCACTGTCCGCTGCTTCGGATT	qRT-PCR
RAD17	GCGGGGCGGGTTGTGGATTT	AGGCACCGGCTGACTGTGGA	qRT-PCR
RAD51	CGCCATTTCCCTCCACTCTCAAGC	ACCTGCTGCCTGAAGCTGTTCG	qRT-PCR
RAD54	TGAGAGACAGGTGGGCACTCC	ACGTCACCTCGTCACCTGCTGA	qRT-PCR
SMC6A	ACCCTTCCTCCCCGTCCTCG	TGCGTTGCTTCTTCAGTCTGCG	qRT-PCR
SMC6B	AGACCTTCGCGACTCTGTGCT	GCGGTGCTTCTTCAGTTGGCG	qRT-PCR
LIG4	GCTGCTGAGGTATTGCAACG	TCTCCGCTCTGTTCTCACTTG	qRT-PCR
REV7	ATTAAACCGTCTTGCGCTGC	ACCCACTTGAGGAAGTGACC	qRT-PCR
PARG1	CGGATGGATGACAATGAAGCT	ATGTACTCACCAGCAAACCGAAA	qRT-PCR
PARG2	TTTGTTTCTTATCCCAAGGCTGAT	CTTCTATAGCTCCCGAGGTGTGA	qRT-PCR
Actin2	ATCGGTGGTTCCATTCTTGCTTC	TGGACCTGCCTCATCATACTCG	qRT-PCR
PARG1	TTTGTAGGATGATTCCAACCG	CGGAGGTGGTTCCCTAAGTAG	*parg1-4* confirmation
PARG1	AATCCTGATTGAGGCATGTTG	ATAAAAGCACCTGGGAAGCAG	*parg1-2* confirmation
LBb1	GCGTGGACCGCTTGCTGCAACT		T-DNA border primer
PARG1	ACGCAAGATTACCGCTGCTCCT	TCGGTGTGACGCAGTAGTTTCTGT	RT-PCR
PARG2	GAGCCACCATGAGTTGGATT	TGCAGCTCTTCTTGCGTGTTCC	*parg2-1* confirmation
GKLB	CCCATTTGGACGTGAATGTAGACAC		T-DNA border primer
PARG2	ATATGCGTCACTGCACGAAG	GGTAGACAGTGAGGTCATGAGCC	RT-PCR
EF-1a	ATGCCCCAGGACATCGTGATTTCAT	TTGGCGGCACCCTTAGCTGGATCA	RT-PCR
PARG1	AACTCCTCGGCGACCGCAAG	CTGCACAATGCAGGAACCACCTCA	Complementation
PARP1	TAAAACCAGAAACATCTACAACGCC	GTTCGTTTACTCTTTTGTGTCGCAT	*PARP1* CDS cloning
PARG1	GCGGCAGCAGAATCTTGTCGC	GGCGGCTGGATAGCTTTGTTGGT	*PARG1* CDS cloning
PARG2	ATGGAACTGAGGGCAGATCT	CTAGGTAGACAGTGAGGTCAGA	*PARG2* CDS cloning

Table S2. Primer list for DNA constructs.

Construct	Forward Primer (5'-3')	Reverse Primer (5'-3')
pET32a-PARP1	GGAGCTCATGGCAAGCCCACATA	GGCGGCCGCTCATCTCTTGTGCTTA
pGEX-4T1-PARG1	CGGAATTCGAGAATCGCGAAGAGCTTAACTC	CGGTCGACTCAAGGCGGCTGGATAGCTTTGTTGGT
p35S-Fast-dsPARG2	1.GAATTCCTGATTCGTGGGCTAAT<br>2.CTGCAGCTGATTCGTGGGCTAATT	1.GGTACCATTGATGCATGTATCCG<br>2.GGATCCATTGATGCATGTATCCG
pZP221-*PARG1*	CGCCTGCAGTTTAATTAGAGAAAGTTTC	CGCGGTACCCCTTATTGCCTGAAAAGGA
pAKK687-GUS	GCGGCCGCAACTCCTCGGCGACCGCAAG	TCTAGATTTTCGATTTTTCTAATCTC
